# Recombinant *S. cerevisiae* expressing Old Yellow Enzymes from non-conventional yeasts: an easy system for selective reduction of activated alkenes

**DOI:** 10.1186/1475-2859-13-60

**Published:** 2014-04-25

**Authors:** Diego Romano, Martina Letizia Contente, Francesco Molinari, Ivano Eberini, Erica Ruvutuso, Cristina Sensi, Alberto Amaretti, Maddalena Rossi, Stefano Raimondi

**Affiliations:** 1 Dipartimento di Scienze della Vita, Università degli Studi di Modena e Reggio Emilia, Via G. Campi 183, 41125 Modena, Italy; 2 Department of Food, Environmental and Nutritional Sciences (DeFENS), University of Milan, Via G. Celoria 2, 20133 Milan, Italy; 3 Dipartimento di Scienze Farmacologiche e Biomolecolari, Università degli Studi di Milano, Via Trentacoste 2, 20134 Milan, Italy

## Abstract

**Background:**

Old Yellow Enzymes (OYEs) are flavin-dependent enoate reductases (EC 1.6.99.1) that catalyze the stereoselective hydrogenation of electron-poor alkenes. Their ability to generate up to two stereocenters by the *trans-*hydrogenation of the C = C double bond is highly demanded in asymmetric synthesis. Isolated redox enzymes utilization require the addition of cofactors and systems for their regeneration. Microbial whole-cells may represent a valid alternative combining desired enzymatic activity and efficient cofactor regeneration. Considerable efforts were addressed at developing novel whole-cell OYE biocatalysts, based on recombinant *Saccharomyces cerevisiae* expressing OYE genes.

**Results:**

Recombinant *S. cerevisiae* BY4741∆*Oye2* strains, lacking endogenous OYE and expressing nine separate OYE genes from non-conventional yeasts, were used as whole-cell biocatalysts to reduce substrates with an electron-poor double bond activated by different electron-withdrawing groups. Ketoisophorone, α-methyl-*trans-*cinnamaldehyde, and *trans-*β-methyl-β-nitrostyrene were successfully reduced with high rates and selectivity. A series of four alkyl-substituted cyclohex-2-enones was tested to check the versatility and efficiency of the biocatalysts. Reduction of double bond occurred with high rates and enantioselectivity, except for 3,5,5-trimethyl-2-cyclohexenone. DFT (density functional theory) computational studies were performed to investigate whether the steric hindrance and/or the electronic properties of the substrates were crucial for reactivity. The three-dimensional structure of enoate reductases from *Kluyveromyces lodderae* and *Candida castellii*, predicted through comparative modeling, resulted similar to that of *S. cerevisiae* OYE2 and revealed the key role of Trp116 both in substrate specificity and stereocontrol. All the modeling studies indicate that steric hindrance was a major determinant in the enzyme reactivity.

**Conclusions:**

The OYE biocatalysts, based on recombinant *S. cerevisiae* expressing OYE genes from non-conventional yeasts, were able to differently reduce the activated double bond of enones, enals and nitro-olefins, exhibiting a wide range of substrate specificity. Moreover whole-cells biocatalysts bypassed the necessity of the cofactor recycling and, tuning reaction parameters, allowed the synthetic exploitation of endogenous carbonyl reductases. Molecular modeling studies highlighted key structural features for further improvement of catalytic properties of OYE enzymes.

## Background

Fine chemical industry is increasingly taking advantage of biocatalysts to produce high-value products because enzymes and whole-cell biocatalysts can be sometimes more convenient than synthetic chemistry [1]. Biotransformations occur under mild conditions of temperature, pressure and pH, and may display their activity with high chemo-, regio- and stereoselectivity [1,2]. Thus, biocatalysis has developed from a niche technology to a widely used manufacturing method, and the availability of new biocatalytic processes for the preparation of optically pure molecules is very attractive for organic chemists.

Due to the increasing demand of optically pure building blocks, considerable efforts are addressed at identifying, characterizing, improving, and developing Old Yellow Enzymes (OYE) biocatalysts, i.e. flavin-dependent enoate reductases (EC 1.6.99.1), that catalyze the chemo- and stereoselective hydrogenation of electron-poor alkenes [3,4]. Their biotechnological potential lies on the ability to generate up to two stereocenters by the stereoselective *anti*-hydrogenation of the activated C = C double bond, which is highly demanded in asymmetric synthesis, since conventional chemical methods mostly give *syn*-hydrogenation [5,6]. OYEs have been found in plants, bacteria, and fungi [7-10], OYE1 from *Saccharomyces pastorianus* being the first to be widely studied for structure, substrate selectivity and stereoselectivity [11-14]. In order to improve the tools for asymmetric reductions of activated olefins, the biodiversity of non-conventional yeasts was explored as well, and yielded several novel OYE genes [15,16]. OYE genes from unconventional yeasts were cloned and expressed in *Saccharomyces cerevisiae* which was used as a whole-cell biocatalyst, demonstrating the important role of unconventional yeasts as sources of novel biocatalysts and their potential for the expansion of known genetic biodiversity [16].

In the present study, recombinant strains of *S. cerevisiae* lacking endogenous OYE and expressing eight OYE genes from as many unconventional yeasts were used as whole-cell biocatalysts to reduce different substrates with an activated electron-poor double bond, in order to compare their catalytic and selectivity properties. It was investigated whether the electronic properties and the steric hindrance affected reactivity and/or enzyme stereoselectivity in the asymmetric reduction. The structure of three selected enzymes was predicted through comparative modeling and the active sites compared. The reactivity of the tested substrates, has been also studied by means of electronic computations, such as the lowest unoccupied molecular orbitals (LUMO) distribution and OYE-substrate interactions were analyzed to reveal the key features of the structure and the sequence patterns of these new enoate reductases.

## Results and discussion

### Asymmetric reductions with recombinant strains of *S. cerevisiae* expressing OYE genes

**Figure 1:**
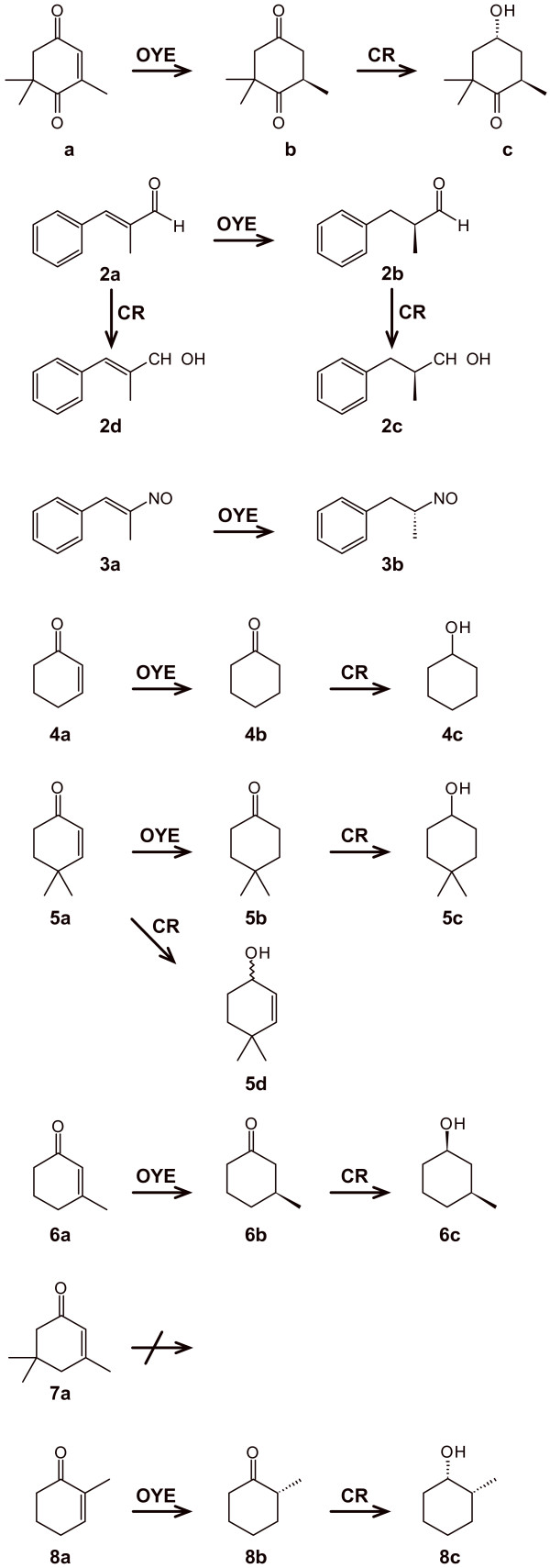
Tested substrates and main products observed during bioreductions.

The strain *S. cerevisiae* BY4741∆*Oye2*, that lacks endogenous *Oye2* gene and presents negligible overall OYE activity [16], was used as the host to express the OYE genes from *S. cerevisiae* BY4741 (*OYE2*), *S. cerevisiae* L12, and from the unconventional yeasts *Candida castellii* DBVPG3704, *Candida sake* DBVPG6162, *Kazachstania exigua* L10, *Kazachstania lodderae* DBVPG6308, *Kazachstania spencerorum* DBVPG6748, *Nakaseomyces bacillisporus* DBVPG6945, and *Naumovia castellii* DBVPG6298 [16]. Recombinant strains bearing heterologous OYE genes were used as whole-cells biocatalysts to reduce three molecules with electron-poor double bond activated by different electron-withdrawing groups (Figure 1), i.e. ketoisophorone (2,6,6-trimethylcyclohex-2-ene-1,4-dione, **1a**), α-methyl-*trans-*cinnamaldehyde ((*E*)-2-methyl-3-phenyl-2-propenal, **2a**), and *trans-*β-methyl-β-nitrostyrene ((*E*)**-**1-phenyl-2-nitropropene, **3a**).

The activity towards **1a** of the diverse recombinant *S. cerevisiae* was evaluated using resting cells. The conversion was at least 86% after 5 h and, for most of the strains, was nearly complete after 24 h (Table 1). However, even though high efficiency of **1a** transformation was observed, and the reaction proceeded with preference for the *R*-levodione (**1b**), the enantioselectivity generally decreased compared to that obtained with the corresponding wild type yeasts [16].

**Table 1:** **Bioreductions of ketoisophorone (1a), α-methyl-*trans*-cinnamaldehyde (2a), and** ***trans*-β-methyl-β-nitrostyrene (3a), with recombinant** ***S. cerevisiae*** **BY4741**∆ ***Oye2*** **bearing heterologous OYE genes**

**OYE gene source**	1a → 1b*			**2a → 2c**			**3a → 3b**		
	**Time**	**1b**	**(*R*)-1b**	**Time**	**2c**	**(*S*)-2c**	**Time**	**3b**	**(*R*)-3b**
	**h**	**%**	**e.e.%**	**h**	**%**	**e.e.%**	**h**	**%**	**e.e.%**
*C. castellii* DBVPG3704	5	≥ 99	70	24	70^†^	≥ 98	2	45	50
*C. sake* DBVPG6162	5	97	71	3	100	59	0.5	93	48
*K. exigua* L10	5	94	82	24	100	≥ 98	0.5	97	30
*K. lodderae* DBVPG6308	5	≥ 99	78	16	100	80	0.5	97	58
*K. spencerorum* DBVPG6748	5	96	65	3	100	≥ 98	0.5	94	50
*Nak. bacillisporus* DBVPG6945	5	98	68	2	100	95	0.5	92	40
*Nau. castellii* DBVPG6298	5	93	78	2	100	85	5	92	29
*S. cerevisiae* BY4741 (Oye2)	5	89	80	2	100	≥ 98	1	96	50
*S. cerevisiae* L12	5	86	79	2	100	72	2	95	44

Data are reported as molar conversion (%). *Results from Raimondi et al. 2010. ^†^30% of compound 2d was also observed. The experiment were performed in triplicate, the yield standard deviations (SD) were always less than 4%, and the ee% SD were always less than 3%.

The biotransformation of **2a** by whole-cells of wild-type Baker’s yeast has been reported to result in a mixture of saturated 2-methyl-3-phenylpropanol (**2c**) and unsaturated primary alcohol 2-methyl-3-phenyl-2-propenol (**2d,** Figure 1), with different ratios depending on the strain and the experimental conditions [17,18]. Consistently, the transformation of **2a** using the wild type *S. cerevisiae* BY4741 gave a mixture of **2c** (78%) and **2d** (22%). On the contrary, *S. cerevisiae* BY4741∆*Oye2*, that lacks endogenous *Oye2* gene, yielded **2d** as the sole detectable product, indicating the pivotal role of OYE2 protein in the enoate reductase activity of the parental strain [19]. Reduction of **2a** with most of the recombinant strains gave **2c** as the only observed product by means of the subsequent C = C and carbonyl reductions (Table 1). The enhanced OYE activity of the recombinant strains prevented the formation of **2d** observed using *S. cerevisiae* BY4741, with the exception of the strain expressing the *C. castellii* OYE gene that yielded a mixture of **2c** and **2d**. Interestingly, enantioselectivity ranged between 59 and > 98%, with four strains yielding (*S*)-**2c** as the sole detectable enantiomer.

The majority of recombinant strains exhibited a high rate in reducing **3a** to 1-phenyl-2-nitropropane (**3b**), but low e.e. (29-58%) was generally observed (Table 1). Similar behavior was observed with different microbial whole-cells, where the scarce enantioselectivity was mostly due to the easy racemization of the saturated nitro-derivative [20].

The data herein reported proved that the OYE biocatalysts, based on recombinant *S. cerevisiae* BY4741∆*Oye2* expressing OYE genes from non-conventional yeasts, were able to efficiently convert not only the enone **1a**, a well-established OYE substrate, but present a broader substrate specificities being able to reduce also the activated C = C double bond of enals (**2a**) and nitro-olefins (**3a**), showing that these systems seem to have a great potential for synthetic chemists.

### Bioconversion of a series of alkyl-substituted cyclohex-2-enones

Recombinant strains were tested to reduce four alkyl-substituted cyclohex-2-enones (**4a**, **5a**, **6a**, and **7a**; Figure 1). The substrates were selected in order to investigate the versatility and efficiency of these biocatalysts, defining how the electronic properties and steric hindrance affect substrate reactivity and enzyme stereoselectivity in the asymmetric reduction of double bond.

All the recombinant yeasts totally reduced 2-cyclohexenone (**4a**) into cyclohexanone (**4b**) within 2 h (Table 2), yielding no traces of cyclohex-2-enol or cyclohexanol (**4c**), that could result from carbonyl reductases (CR) activity on the keto group of **4a** or **4b**, respectively.

**Table 2:** **Bioreductions of alkyl-substituted cyclohex-2-enones (substrates 4a, 5a, 6a, and 7a**^**#**^**) with recombinant** ***S. cerevisiae*** **BY4741**Δ ***Oye2*** **bearing heterologous OYE genes**

**OYE gene source**	**4a → 4b**		**5a → 5b**		**6a → 6b**^**‡**^		
	**Time**	**Yield**	**Time**	**Yield**	**Time**	**Yield**	**(*S*)-6b**
	**h**	**%**	**h**	**%**	**h**	**%**	**e.e.%**
*C. castellii* DBVPG3704	2	≥ 99	2	76^†^	72	< 5	n.d.
*C. sake* DBVPG6162	1	≥ 99	1	95	48	75	≥ 98
*K. exigua* L10	2	≥ 99	2	92	72	23	≥ 98
*K. lodderae* DBVPG6308	2	≥ 99	2	84^†^	72	25	≥ 98
*K. spencerorum* DBVPG6748	2	≥ 99	1	95	48	60	≥ 98
*Nak. bacillisporus* DBVPG6945	1	≥ 99	2	94	72	24	≥ 98
*Nau. castellii* DBVPG6298	2	≥ 99	2	95	48	53	≥ 98
*S. cerevisiae* BY4741 (Oye2)	1	≥ 99	2	96	48	45	≥ 98
*S. cerevisiae* L12	2	≥ 99	1	97	48	40	≥ 98

Data are reported as molar conversion (%). ^#^No conversion of compound **7a** was observed in the first 72 h. ^†^15-20% of compound **5d** was also observed; ^‡^product **6c** was always less than 15%. The experiment were performed in triplicate, the yield standard deviations (SD) were always less than 4%, and the ee% SD were always less than 3%.

The strains also reduced 4,4-dimethyl-2-cyclohexenone (**5a**) into 4,4-dimethyl-cyclohexanone (**5b)** at a good extent, with yields ranging between 76 and 97%. The strains carrying the genes from *Candida castellii* DBVPG3704 and *Kazachstania lodderae* DBVPG6308 showed the lowest conversions and produced also 4,4-dimethyl-2-cyclohexenol (**5d**; 20% and 15%, respectively) through carbonyl reduction of **5a**, indicating that CR became competitive when C = C double bond reduction was less effective.

The yeast bearing the OYE gene from *C. castellii* DBVPG3704 was the only ineffective in the reduction of 3-methyl-2-cyclohexenone (**6a**). All the other strains reduced this substrate with high stereoselectivity (>98%), with homogeneous stereobias and formation of (*S*)-**6b** as the sole detectable enantiomer. Small amounts of 3-methylcyclohexanol (**6c**, 3-6%) were observed after 48 h. Conversion yield of **6a** towards **6b** ranged from 23 to 75% after 72 h and was lower compared to the conversion of substrates **4a** and **5a**. Consistently, previous studies proved that **6a** is a ‘difficult’ substrate for enoate reductases, due to the presence of a substituent on the C3 position [21,22]. All the recombinant strains failed to transform α-isophorone (3,5,5-trimethyl-2-cyclohexenone, **7a**), neither the C = C or C = O double bonds being reduced after 72 h.

As a whole, most of the nine recombinant strains exhibited similar activity toward the tested cyclohexenone derivatives and a common stereobias was observed toward **6a**. Moreover, substrates **4a**, **5a**, **6a** and **7a** which are increasingly sterically hindered, were progressively less reactive in C = C reduction. The electronic features of the tested substrates and the structural properties of the investigated OYEs will be thoroughly described in a following section.

### Computational analysis: evaluation of substrate electronic features, and OYE three-dimensional structure prediction through comparative modeling

**Figure 2:**
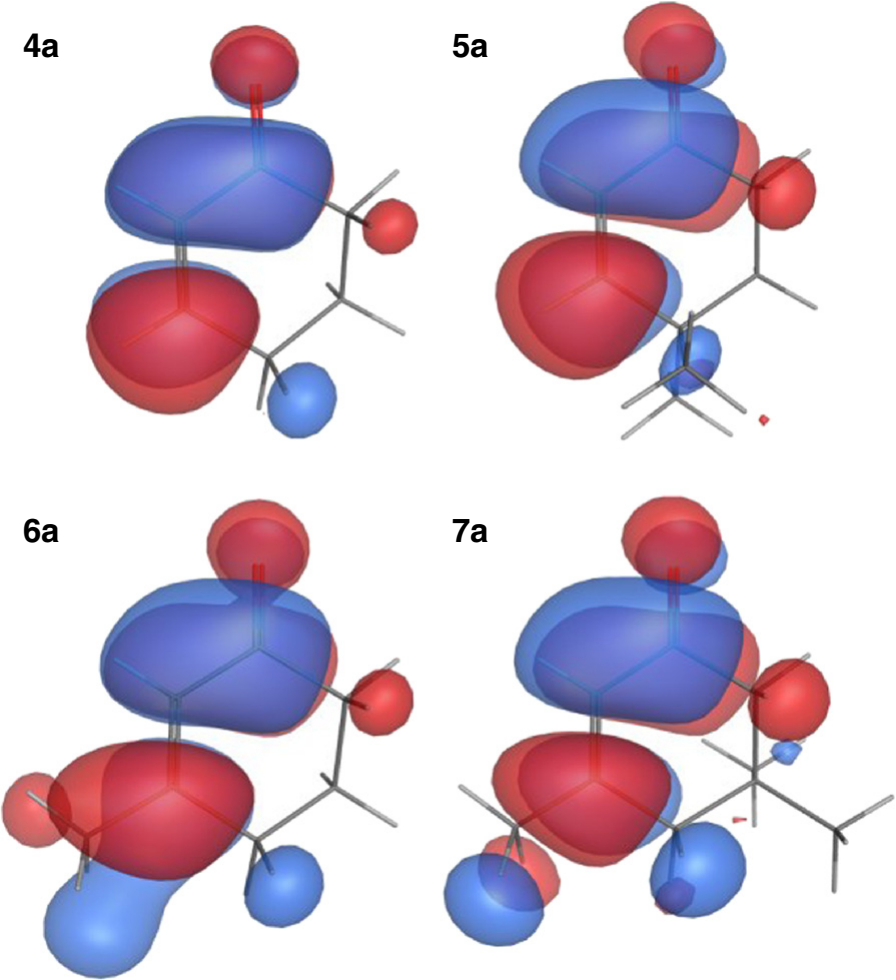
Lower Unoccupied Molecular Orbitals (LUMO) of the alkyl-substituted cyclohex-2-enones.

The enoate reductases preferentially reduce substrates containing electron-poor double bonds [3]. In order to evaluate the electronic features of the tested substrates, a geometry optimization in the gas phase with an approach based on density functional theory (DFT/B3LYP/6-31G*) was performed, resulting in prediction of the double bond lengths and of the electrostatic potential (ESP) derived charges for the C atom involved in the hydride attack of the substrates (Table 3). A further characterization has been carried out by the analysis of the lowest unoccupied molecular orbitals (LUMO) for the investigated substrates. The surfaces were rendered in order to encapsulate approximately 80% of the integral of the evaluated grid points (Figure 2). Based on the length of the double bonds, **4a**, **5a**, **6a**, and **7a** resulted very similar. The values 1.34 Å for substrates **4a** and **5a** and 1.35 Å for substrates **6a** and **7a**, typical of the sp^2^ hybridization state, were not sufficiently different to explain a diverse reactivity based on electronic configurations. The comparison among the LUMO frontier orbitals revealed very similar configuration and reactivity around the sp^2^ carbons of all the substrates. Conversely, computed ESP charges suggested that substrates **5a**, **6a**, and **7a** were the most favored for the reduction of the double bond, outcome partially in contrast with bioconversion data indicating **6a** and **7a** as “difficult” substrates.

**Table 3:** Electronic features of the alkyl-substituted cyclohex-2-enones predicted by DFT calculations

**Substrate**	**Double bond length**	**ESP charge**
**4a**	1.34 Å	-0.003
**5a**	1.34 Å	-0.174
**6a**	1.35 Å	0.350
**7a**	1.35 Å	0.404

The data herein presented demonstrated that the different reactivity of these substrates cannot be ascribed to their electronic configurations. As a whole, the substrates which are increasingly sterically hindered, were progressively less reactive in double bond reduction and their electronic properties do not affect this behavior.

**Figure 3:**
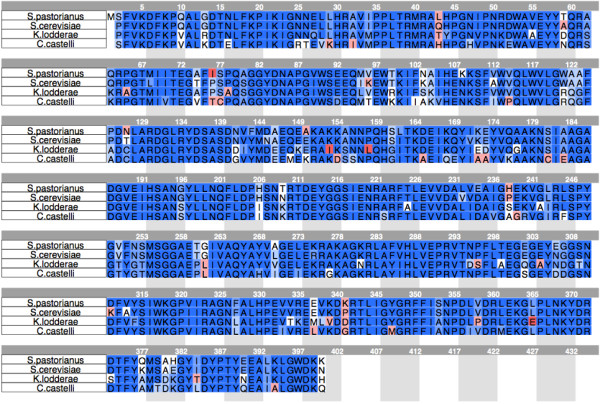
**Sequence alignment of OYE proteins.** Sequence alignment among the template 1OYA from *S. pastorianus* and the three OYE proteins selected for the structure prediction through comparative modelling.

In order to evaluate the role of steric hindrance with respect to the substrate selectivity, the three-dimensional structures of the *S. cerevisiae* OYE2 and of the *K. lodderae* DBVPG6308 and *C. castellii* DBVPG3704 enoate reductases were predicted through comparative modeling, using the structure of the *S. pastorianus* OYE1 (RCSB PDB: 1OYA) as template [11]. These three enzymes were chosen because, when overexpressed in *S. cerevisiae* BY4741∆*Oye2*, the corresponding recombinant strains exhibited different activity and/or enantioselectivity towards many of the tested substrates. The proteins and the template OYE1 share a very high level of identity, ranging from 73 to 91% (Table 4, Figure 3). The active sites are highly conserved and few differences in amino acid composition are present (Table 5). Furthermore, similar distances between the three core amino acids (His191, Asn194, and Tyr196) belonging to the catalytic site have been calculated (Table 6). The high identity between both the primary structures of the investigated OYEs and the low root mean square deviation (RMSD) values for the modeled structures suggested that the different transformation rates of the tested substrates can hardly be ascribed only to the structural diversity elucidated by our computational work.

**Table 4:** OYE sequences alignment

	**1OYA**	**OYE2**	**OYE from** ***K. lodderae***	**OYE from** ***C. castellii***
1OYA	-	91.0	77.2	74.8
OYE2	91.0	-	77.7	73.2
OYE from *K. lodderae*	77.2	77.7	-	77.4
OYE from *C. castellii*	74.8	73.2	77.4	-

Sequence identity (%) among the template 1OYA and the three OYE proteins selected for the structure prediction through comparative modelling.

**Table 5:** Amino acid composition of OYE active sites

**Position**	**OYE2**	**OYE from *K. lodderae***	**OYE from *C. castellii***
82	TYR	TYR	TYR
116	TRP	TRP	TRP
117	VAL	VAL	VAL
118	LEU	LEU	LEU
119	GLY	GLY	GLY
**120**	**TRP**	**ARG**	**ARG**
**121**	**ALA**	**GLN**	**GLN**
**122**	**ALA**	**GLY**	**GLY**
191	HIS	HIS	HIS
192	SER	SER	SER
193	ALA	ALA	ALA
194	ASN	ASN	ASN
**195**	**GLY**	**SER**	**SER**
196	TYR	TYR	TYR
197	LEU	LEU	LEU
198	LEU	LEU	LEU
199	ASN	ASN	ASN
200	GLN	GLN	GLN
201	PHE	PHE	PHE
**250**	**PHE**	**TYR**	**TYR**
**251**	**ASN**	**GLY**	**GLY**
253	MET	MET	MET
**295**	**PRO**	**SER**	**PRO**
296	PHE	PHE	PHE
375	TYR	TYR	TYR

Differences are highlighted in bold.

**Table 6:** OYEs catalytic triad

**Protein**	**His-Asn**	**His-Tyr**	**Tyr-Asn**
*OYE2*	6.5	6.8	5.1
*K. lodderae*	7.5	6.0	5.3
*C. castellii*	7.3	5.6	5.5

Distances between the core amino acids belonging to the catalytic site (His191, Asn194, and Tyr196) in the three modelled OYEs (Å).

**Figure 4:**
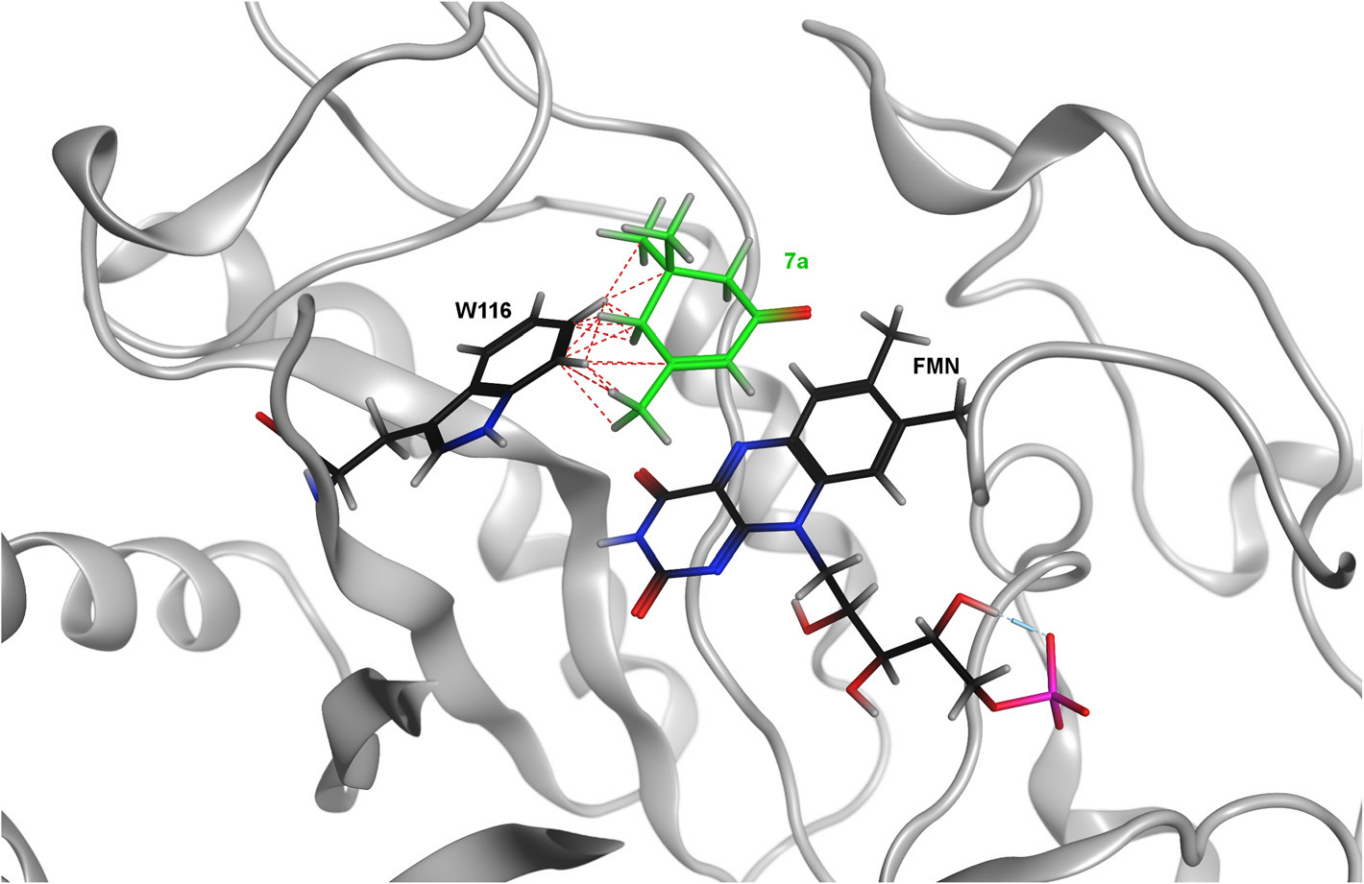
Repulsive van der waals interactions between the alkyl groups of α-isophorone (7a) and OYE2 Trp116.

The substrate α-isophorone (**7a**) was not reduced by OYEs, although electronic features suggested a good reactivity, likely because of a dramatic steric effect. A docking study of this molecule have been performed on the predicted structure of OYE2 protein. The repulsive van der Waals interactions between the alkylic groups of substrate **7a** and Trp116 of OYE2 confirmed that steric hindrance was present (Figure 4). Consistently, in a previous study, the interactions between substrates and Trp116 in the active site of *S. pastorianus* OYE1 influenced the catalytic activity of the enzyme and the stereochemistry of the products [23].

The analysis of the unfavorable contacts between the side chain of Trp116 and the substrates confirmed that the transformation efficiency on a series of alkyl-substituted cyclohex-2-enones was associated to the length of the alkyl-substituent. The larger was the substrate hindrance, the lower was the reactivity. Furthermore, the pivotal role of Trp116 on catalytic properties of OYE enzymes has been highlighted, suggesting this key structural features as an hot spot for modification and further improvement of the biocatalysts.

### Whole-cell OYE biocatalysts: synthetic exploitation tuning reaction time

**Figure 5:**
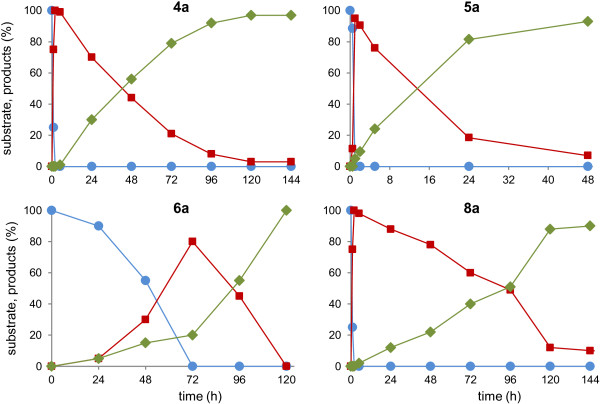
**Bioreductions of alkyl-substituted cyclohex-2-enones (substrates 4a, 5a, 6a, and 8a) until prolonged reaction times.** Data are reported as molar conversion (%). Blue line and circles: substrates. Red line and squares: OYE products; Green line and diamonds: OYE and CR products. The curves were representative of three independent experiments and the standard deviations were always less than 4%.

Biotransformation of αβ-unsaturated carbonyls occurred with carbonyl reduction by endogenous CR as side-reaction. Thus, the biotransformations of substrates **4a**, **5a**, **6a**, and **8a** were analyzed until prolonged reaction times, in order to obtain also the reduction of the carbonyl group. In all cases we observed a prevailing OYE activity in the first hours of reaction, while at longer times the carbonyl reductase activity led to the formation of the saturated alcohols (Figure 5). More in detail, using **4a** as substrate, it was possible to quantitatively recover **4b** after 2 hours of reaction. Later (144 h), the endogenous carbonyl reductase(s) led to the formation of **4c,** with high yield (97%). The same behavior was observed with substrate **8a**: at short reaction times (2 h) the OYE product (*R*)-**8b** was almost (99%) the only chemical species present, whereas a 1/10 ratio betwee**n 8b** and **8c** was observed after 168 h; the stereochemical analysis of **8c** showed that the ratio (1*S*,2*R*)-**8c**/(1*R*,2*R*)-**8c** was 70/30. The bioreduction of **6a**, that is a less reactive substrate, proceeded with the concomitant formation of **6b** and **6c**; **6b** was obtained with high enantioselectivity (e.e. > 98%). The saturated alcohol (**6c**) was the minor product during the first hours of reaction and became the sole product after 120 h; **6c** was produced with a *syn/anti* composition of 75/25. In the case of **5a**, bioreductions proceeded faster: the saturated ketone **5b** was produced at 95% molar conversion after 1 h while **5c** reached 95% after 48 h of biotransformation.

Overall, our whole-cells system, compared to the use of purified enzymes, showed a peculiar synthetic advantage: with a unique biocatalyst, tuning the reaction time, it is possible to have access to different molecules, with high yields, starting from a single substrate.

## Conclusions

*S. cerevisiae* is well suited for biotechnological applications owing to the wealth of the genetic techniques available, the ease of manipulation and the rapid growth rates. The expression in the host *S. cerevisiae* BY4741∆*Oye2* of the OYE genes make possible the exploitation of these technological feature leading to the development of efficient biocatalysts base on yeast cells. The recombinant *S. cerevisiae*, showed a wide range of substrate specificity, being able to reduce the activated C = C double bond of enones, enals and nitro-olefins. Moreover, whole-cells biocatalysts bypassed the necessity of the cofactor recycling and, tuning reaction parameters, allowed the synthetic exploitation of the endogenous carbonyl reductases. Versatility and efficiency of these biocatalysts was analyzed revealing that the different reactivity was not ascribed to substrate electronic properties, but steric hindrance resulted the most relevant factor affecting the asymmetric reduction of double bond. Molecular modeling studies on OYE proteins highlighted the pivotal role of Trp116 both in activity and stereochemistry and it was established as a key structural features for further improvement of catalytic properties of OYE enzymes.

## Methods

### Strains and culture conditions

Recombinant *S. cerevisiae* BY4741∆*Oye2* strains (bearing OYE genes from *Candida castellii* DBVPG3704, *Candida sake* DBVPG6162, *Naumovia castellii* DBVPG6298, *Kazachstania exigua* L10, *Kazachstania lodderae* DBVPG6308, *Kazachstania spencerorum* DBVPG6748, *Nakaseomyces bacillisporus* DBVPG6945, *Saccharomyces cerevisiae* BY4741 (Oye2), and *Saccharomyces cerevisiae* L12) were prepared as described earlier [16]. Strains were routinely maintained on YNB agar medium (DifcoTM Yeast Nitrogen Base without aminoacids 6.7 g/L, glucose 20 g/L, histidine 0.15 g/L, leucine 0.73 g/L, methionine 0.15 g/L) and grown in YNB liquid medium, in baffled Erlenmeyer flasks on orbital shaker at 30°C and 150 rpm.

### Biotransformations

Recombinant cells grown for 24 h were centrifuged (5000 rpm for 10 min at 4°C), washed and resuspended at a concentration of 15 AU of OD_600_ in 100 mM phosphate buffer at pH 7, containing 50 g/L glucose. Substrate stock solutions (200 g/L) prepared in ethanol were added to the reaction obtaining 1 g/L of substrate concentration. Biotransformations were carried out in baffled Erlenmeyer flasks on orbital shaker, incubated at 30°C and 150 rpm. Samples for analysis were extracted 1:1 with EtOAc containing 0.05% (v/v) of 1-phenylethanol as internal GC standard and dried over Na_2_SO_4_. At the end of the biotransformation, the whole reaction mixture was centrifuged and the aqueous supernatant extracted three times with EtOAc. The combined organic phases were dried over Na_2_SO_4_ and the residue purified by flash chromatography.

### Analytical methods

Gas chromatographic analysis were performed on a Dani 86.10 HT instrument (Dani Instruments, Italy; carrier H_2_, 0.6 bar, FID detector), using a MEGA-DEX DET-Beta (column A; Mega Snc, Legnano, Italy) chiral column or a SE30 polydimethylsiloxane non-chiral column (column B), under the following conditions. Bioreduction of **1a**: column A; 45°C for 2 min, 5° min^-1^ rate to 130°C, 30° min^-1^ to 200°C; retention times: **1a** 18.0 min, (*R*)-**1b** 19.2 min, (*S*)-**1b** 19.5 min. Bioreduction of **2a**: column A; 95°C, 1° min^-1^ rate to 120°C, 30° min^-1^ to 200°C; retention times: **2a** 31.0 min, (*R*)-**2b** 16.4 min, (*S*)-**2b** 16.7 min, (*R*)-**2c** 28.7 min**,** (*S*)**-2c** 29.2 min, **2d** 19.4 min. Bioreduction of **3a**: column B; 105°C for 5 min, 1° min^-1^ rate to 115°C, 30° min^-1^ to 200°C; retention times **3a** 20.4 min, (*R*)-**3b** 18.8 min, (*S*)-**3b** 19.0 min. Bioreduction of **4a**: column B; 55°C isotherm analysis; retention times **4a** 5.1 min, **4b** 4.1 min. Bioreduction of **5a**: column B; 60°C for 3 min, 1°C min^-1^ rate to 140°C, 30° min ^-1^ to 200°C; retention times **5a** 14.6 min, **5b** 14.0 min, **5c** 16.9 min, **5d** 16.2 min. Bioreduction of **6a**: column A; 50°C, 1°C min^-1^ rate to 150°, 30° min^-1^ to 200°C; retention times **6a** 20.9 min, (*R*)-**6b** 13.0 min, (*S*)-**6b** 13.7 min. Column B; 40°C isotherm analysis; retention times **6a** 25.3 min, **6b** 11.4 min, **6c** 12.3 min. Bioreduction of **7a**: column A; 60°C for 5 min, 3°C min^-1^ to 100°C, 30° min ^-1^ to 200°C; retention times **7a** 19.5 min. Bioreduction of **8a**: column A; 50°C for 5 min, 5°C min^-1^ rate to 150°, 30° min ^-1^ to 200°C; retention times **8a** 19.8 min, (*R*)-**8b** 12.3, (*S*)-**8b** 13.0. Column B; 50°C for 5 min, 5°C min^-1^ rate to 120°, 30° min ^-1^ to 200°C; retention times **8a** 8.70 min, **8b** 7.02 min, **8c** 7.35 min. Biotransformation products were identified by comparison with authentic reference materials. The absolute configuration and e.e. of **6b** and **8b** were determined by co-injection in chiral GC with reference materials of known absolute configuration [21 and references therein]. Stereoisomeric composition of **6c** and **8c** were determined by ^1^H NMR [24] after purification of the products.

### Computational details

All the comparative modeling procedures were carried out with the Homology module of the Molecular Operating Environment 2011.10 (MOE; Chemical Computing Group Inc., Montreal, QC, Canada). The alignment produced by the Align program of MOE with default parameters was manually adjusted. Comparative model building was carried out with the MOE Homology Model program. 1OYA (*S. pastorianus* OYE1) was set as template for all the modeled sequences. For each sequence, ten independent models were built and refined, and then the highest scoring intermediate model was submitted to a further round of energy minimization (EM). For both the intermediate and the final structures the refinement procedures consisted in EM runs based on the AMBER99 force field, with the reaction field solvation model.

Geometry optimization and electronic computations of the substrates were carried out in the gas phase through the MOE SCF Calculation interface to the GAMESS software [25,26]. The DFT calculations were based on the B3LYP hybrid functional with the 6-31G* basis set.

## Abbreviations

OYE: Old Yellow Enzyme (enoate reductase); CR: Carbonyl reductase; 1a: 2,6,6-trimethylcyclohex-2-ene-1,4-dione [ketoisophorone]; 1b: 2,2,6-trimethylcyclohexane-1,4-dione [(6*R*)-levodione]; 1c: 4-hydroxy-2,2,6-trimethylcyclohexaone [(4*R*, 6*R*)-actinol]; 2a: (*E*)-2-methyl-3-phenyl-2-propenal [α-methyl-*trans*-cinnamaldehyde]; 2b: 2-methyl-3-phenylpropanal; 2c: 2-methyl-3-phenylpropanol; 2d: 2-methyl-3-phenyl-2-propenol; 3a: (*E*)**-**1-phenyl-2-nitropropene [*trans*-β-methyl-β-nitrostyrene]; 3b: 1-phenyl-2-nitropropane; 4a: 2-cyclohexenone; 4b: Cyclohexanone; 4c: Cyclohexanol; 5a: 4,4-dimethyl-2-cyclohexenone; 5b: 4,4-dimethylcyclohexanone; 5c: 4,4-dimethylcyclohexanol; 5d: 4,4-dimethyl-2-cyclohexenol; 6a: 3-methyl-2-cyclohexenone; 6b: 3-methylcyclohexanone; 6c: 3-methylcyclohexanol; 7a: 3,5,5-trimethyl-2-cyclohexenone [α-isophorone]; 8a: 2-methyl-2-cyclohexenone; 8b: 2-methylcyclohexanone; 8c: 2-methylcyclohexanol; DFT: Density functional theory; ESP: Electrostatic potential; LUMO: Lowest Unoccupied Molecular Orbitals.

## Competing interests

The authors declare that they have no competing interests.

## Authors’ contributions

DR and SR conceived the study, performed the experimental work and drafted the manuscript. MC performed bioreductions and chemical analysis. ER and CS contributed to the computational analysis. AA, FM, IE and MR accomplished data interpretation, participated in the design of the study, and writing of the manuscript. All authors read and approved the final manuscript.
